# Kate Mountstephen

**Published:** 1887-08-27

**Authors:** C. G. Furley


### Kate Mountstephen.

By C. G. Furley.

### Chapter I.—The Test of Time.

He knew the monogram, he knew the shape of the envelope, he knew the faint perfume that still lingered about it, in spite of its tossing among plebeian missives in the post-bag; yet he put it unread into the pocket of his coat, and hurried off to the dog-cart that was waiting to carry him and his host to the station, *en route* for a rather distant meet of the most famous hounds in Foxshire. Six months ago the sight of that "E. E.," with the letters snakily intertwisted, had made his pulses throb faster, and let who would wait, there was no delay in opening the little notes in which Evelyn Ellis informed him of her engagements for the next day or two, in order that some carefully arranged accident might bring him to her side. But that was six months ago, when he was meeting her every day, and had no time to forget the charm of her golden hair, and the long-lashed dark eyes which contrasted so effectively with it. Time tries all things; among others, it tries whether a man's love for a beautiful woman is love for her beauty or for herself—a distinction with a considerable difference.

Harry Barnett's passion had not stood the test. He had not yet admitted to himself that he had ceased to love Miss Ellis; but she had certainly receded more completely into the background of his mind than he had thought possible on the day they parted. That was in July, it was now January; and though he would not have avowed it, he had been more chilled than he liked to confess by the calm way in which she had looked upon their separation as final and inevitable.

"It's no use, Harry. Oh yes, I love you; you may be assured of that, but it doesn't affect the point at issue. We are both poor, far too poor to think of marrying each other. Yes, I know you would risk it, and I know, too, that you aren't one of those reckless fellows who go on living on nothing a year, and getting into debt, and finally bring their wives to ruin; but mamma would never permit it. You don't know mamma."

The last sentence was a favourite one of Evelyn's. To Harry Barnett, as to the world at large, Mrs. Ellis appeared to be a weak-minded woman, worldly indeed in her ideas and aspirations, but by no means of such iron mould as to be able to withstand her daughter's will when the latter chose to exercise it. That was what she seemed; but when Harry ventured to assume that her character was in accordance with her appearance, he was invariably met with the formula, "You don't know mamma"; who, according to Miss Ellis, was a person of inflexible determination behind her meek aspect, and who had made up her mind that her beautiful daughter must marry a rich man.

"She liked you immensely, Harry, while you were your uncle's heir; but that marriage of his put you out of her good graces for ever. There's no hope of her ever relenting unless both he and his baby son die. Then, you know, it would be different."

Harry lost patience to a slight degree. "Women are supposed to be gentler than men," he said; "but the calm way in which they talk of the possible deaths of people who stand in their way is horrible. They don't seem to think that the sixth commandment can be broken in thought, or that it applies to them. They are far more callous than we are."

"But I'm not like that!" returned Evelyn plaintively. "It's mamma who—"

"I know that, my darling; I had no thought of blaming you," he answered; being in truth of an idealising nature, which would see no flaw in his mistress's perfection. "Well, if Mrs. Ellis ever relents, or if you make up your mind to defy her, write me a line, and I'll be at your side within twenty-four hours."

So he had said, and so he meant, as he kissed Evelyn for the last time and went away to do his work, and bear his loss as well and bravely as he could. So far as he was personally concerned he did not think his position by any means a bad one. He would not be so rich as if his uncle the brewer had not married a young wife in his old age. His baby cousin had naturally displaced him—he saw no injustice in that; but his uncle would still help him more or less, and he had youth and health, and a shrewd comprehension of the mysteries of stock-broking, to assist him up the ladder of life. There was no reason why he should not be a rich man yet. He had expressed his hopes to Evelyn, but she only sighed.

"Yes," she said, "you may be rich some day—when you are fifty perhaps, and I am forty-five: but what will be the good of that?"

For Evelyn—that is, Mrs. Ellis—seemed to think it quite out of the question that he should marry on a moderate income, or that she should consent to wait till his prospects brightened. They must part—now and for ever.

Therefore he went back to his desk with the conviction that his heart was broken and his life permanently blighted, while Mrs. Ellis and her daughter went abroad, the better to put an end to this useless love-affair. Harry was quite convinced that he should love Miss Ellis to his dying day; and perhaps had not realised that he was not so very miserable after all, till this morning when he put Evelyn's unexpected letter into his pocket unread, and did not even remember to do as he had intended, take it out and peruse it in the dog-cart or the train.

But there was some excuse for his remissness. He could not have holidays *ad libitum* now, for he was honestly determined to prosper in the world if industry would enable him to do so; and when a man has been asked to a country-house for a fortnight's hunting, and the first week has been spoiled by frost, he may, if he is only five-and-twenty, very reasonably be excited at the prospect of a good run on a good horse across the best of the "happy hunting-grounds" of England. Moreover, the love a woman arouses in a man's heart bears inevitably some relation to her character, and Barnett's passion for Evelyn Ellis had not been marked by that tender reverence which makes anything belonging to the loved one a treasure to be valued for ever. It had been *amour de tête*, admiration of her beauty, that was all: the admiration of a true and honest heart that was ready to assume that so fair a woman must be fair-souled; but not the love of a life, that complete fitness of union in which beauty plays only a secondary part.

He and his host, Captain Mitchell, had a great many things to talk about during their drive, chiefly reiterated hopes that the scent would be good and that their horses would not be frightened by the railway journey which was to begin their day's work; and in the train there were several men in the same carriage who were bound on the same expedition as themselves, and whom Mitchell knew; so it would have looked absurd, even if Harry had thought of doing it, to bring out and read under such circumstances a letter so visibly feminine as Miss Ellis's. And by the time they had reached the meet the epistle was forgotten as completely as if it and its fair writer had never existed.

"Who is that young lady?" Harry asked of his host, pointing out a girl on a tall chestnut horse, who sat calmly and quietly in her saddle, and did not seem to think it necessary to canter to and fro, and smile and flirt, as most of the other ladies did.

"That? Well, you have good eyes, Barnett; you have picked out the most notable personage, in my opinion, who is here to-day. That is Miss Mountstephen."

"And Miss Mountstephen is—"

"Oh! nobody very particular in a society sense, but a very remarkable girl, to my mind. Her father is the chief doctor in Coteswold, the village here, and they say she is as good a surgeon as he—which is doubtless an exaggeration."

"A lady doctor!" said Barnett, in accents of distaste.

"No; at least not a professed and qualified one; only a woman who likes to help her fellow-creatures, and finds a little skill of as much use as a great deal of sympathy. I have met old Mountstephen; he is very proud of his daughter—'my Kate' is continually on his lips—says she has the surgical instinct, and encourages her to follow it in every way. He told me she was particularly happy in managing child-patients, and often persuaded them to submit to small operations at her hands when they wouldn't let him touch them."

"She seems rather exceptional."

"She is—quite a character in her way. She does a good deal of nursing the poor people in the village, I am told, and had an ambulance class for girls last winter. There was a bit of a joke about that, which shows that she has a sharp tongue, among other good qualities. Old Lady Bradstock, who is very High Church, was much taken with the idea of ambulance lectures, but wanted them to be given in connection with some church guild—only communicants to be allowed to attend, and so on. Miss Mountstephen refused to have anything to do with them on these terms, and said to her, 'I am afraid your ladyship doesn't understand anatomy. Really, though you might not think it, the Broad Church, the Evangelicals, and even Dissenters, are liable to exactly the same accidents as pure Anglicans.' The story goes that Lady Bradstock replied, 'You don't say so!' but there may be some inaccuracy about the statement."

"But," said Barnett, in a tone of bewilderment, "she seems to be quite a good-looking girl, this Miss Mountstephen."

"She is," and Captain Mitchell's eyes followed those of his friend, as they rested on the slim, dark figure, which seemed very tiny perched up on the tall hunter. She was now talking to some old ladies who were seated in a carriage near, and they could perceive that the calm alertness, which was the usual expression of her face, could soften into a very fascinating smile. "She is, and she can ride, too. You may see how she goes to-day, and you need not be above taking a lead from her; she knows the country well. But here again is a thing that is characteristic of her. She will stop in the middle of a run and go home, if she thinks that by going on to the end she will be late in returning, and her father will be anxious about her safety."

"That is rather a sacrifice, I should think."

"Not a doubt of it; but she never thinks twice about that."

"You seem to admire her, Mitchell."

Captain Mitchell turned away his head with a slight laugh, not caring to confess that the previous winter he had been at some pains to make his admiration of her obvious to Miss Mountstephen, and that she had refused to marry him. "You would admire her too if you saw a little more of her," he said.

"Not I," said Harry; "she is too unique for my taste, and"—looking critically at the young lady's hair—"I don't care for dark women."

The remark brought to his mind a brief remembrance of golden-haired Evelyn's unread letter; but the meet was hardly a place where he could bring it out and peruse it.

Harry Barnett was a good rider, but he could not be expected to go across country like men who hunted three times a week every winter of their lives, though indeed the most evident difference lay in his superior rashness. The field was large, but Harry succeeded in keeping his eyes on Miss Mountstephen, and in the first run—a brief one—he actually did find himself following her lead.

"Keep well to the left, or you'll get staked," she called out to him, as she went over a hedge in front of him.

When he had followed her into the next field she pulled up her horse a little, and when he had come abreast of her, apologised for warning him.

"I ought not to have done it, I know," she said, with a frank smile; "but I don't think I have seen you out with this pack before, and I, being a native, know every inch of the ground here. I hope you are not so much of a man as to be offended by a woman offering you advice."

"I am very much obliged to you," he answered. "I am here only on a brief visit, and I don't want to damage either my own neck or my friend's horse."

After a smart run across two or three more fields the fox was killed. Miss Mountstephen turned away from the sight of the hounds, as each struggled fiercely to get at the prey.

"It is very inconsistent of me," she said to Harry, who had kept by her side; "but whenever I think of the real object of the sport—the killing of the fox—I detest hunting. And indeed I really don't see why we should need the excitement of slaughter; it would be quite as amusing, and much less cruel, if we merely played at 'follow my leader' across hedges and ditches."

"And yet you ride to hounds yourself," Barnett could not refrain from saying.

"Yes; I am inconsistent, as I said. I like the exhilaration of rushing through the air, and the momentary excitement of leaping, so I take it—at the expense of the fox."

Captain Mitchell approached. "We are going to draw Copsehead spinney, Miss Mountstephen," he said. "Shall we start for it?"

"I won't go farther," she answered. "If you get a fox at Copsehead, which gives you anything of a run, you will be out pretty late; and since he has given up hunting himself, my father always thinks I have met with some accident if I am out after two o'clock."

"I don't think you could be thrown," observed Mitchell; "you and your horse seem one."

"Yet I have known what a fall is—the sensation of the earth seeming to rise up and strike me, while all the sky, and the dogs, and horses whirled above me *in vacuo*,—and the country round Copsehead is pretty stiff. I should not wonder if there were some casualty before the day is done."

"I wish you would come," said Barnett, moved by an indefinable impulse. "I believe I should have come to grief already but for you; don't withdraw your guidance now."

"Captain Mitchell is a safer guide than I; he considers the bearings of every step he takes; while my instinct is to go straight on without thinking of the possible consequences."

"I fancy," said Mitchell, not looking at the girl, and speaking with a quiet seriousness of tone which the occasion did not seem to demand, "I fancy that your motto in all things, great and small, is '*Fais ce que dois, advienne que pourra*.'"

Miss Mountstephen blushed. "You take high ground about a very humble matter," she remarked; "but if that is my motto—I don't know if it is; I never thought about it—you must admit that it has one supreme merit, that of simplicity."

"One sometimes regrets doing one's duty; it doesn't always answer one's expectations."

"I have yet to find that out," said Miss Mountstephen, as she turned away and set out on her homeward way, followed by the grave, elderly groom who attended her.

"What do you think of her?" asked Mitchell as they trotted off towards Copsehead spinney.

"I like her. She is decidedly a girl of 'pluck,' but she is something more than that. She gives one an impression of peculiar reality, of being reliable as well as self-reliant."

"That's just what she is."

"I don't know that that's my ideal of womanhood; I prefer something more dependant."

Mitchell demurred. "Your dependant women are very pretty, pleasant creatures for fair weather; but in such a mixed-up affair as this life of ours it is sometimes good to have a woman on whom one can in turn depend. The world would be a better place than it is if there were more women in it as true, and brave, and capable as Kate Mountstephen."

At this Harry perceived that his friend's regard for the young lady was something more than a mere admiration for a woman who could ride well, and so forbore to express any difference of opinion.

A fox was found at Copsehead—a well-behaved fox, willing to give its pursuers a long and winding run before it met its fate. But it chose also that they should imperil their limbs by jumping the biggest fences and flying headlong over all the brooks in the neighbourhood. Harry Barnett was in front all the time. In fact, he let his horse use himself up rather recklessly, the result being that when towards the end of the run they came to a hedge with a tolerably wide ditch beyond it, the animal landed a foot or two short, and Mr. Barnett was thrown

He fell on his left shoulder. An intense pain shot through him, and when he tried to move his arm it was powerless. He struggled to his feet with the help of his right hand, though the suffering nearly made him faint.

"What has happened, Barnett?" cried Mitchell, hurrying up to him, and inwardly cursing himself for not doing more to restrain his guest's impetuosity.

"I don't know. I think my arm is broken," stammered Harry, with pale lips; but an old huntsman, who had seen the accidents of twenty winters, said, "I think, sir, it's not broken. From the way it hangs, I should think the shoulder's out of joint, which is far more painful than a break."

"What shall we do?" asked Mitchell.

"We aren't far from Coteswold. The best thing we can do is to take the gentleman to Dr. Mountstephen's. He knows every sort of smash a man can come by in the hunting-field, and if your friend isn't able to go home he'll have Miss Kate to take care of him, and that's something worth bearing a bit of pain for."

The voices seemed a long way off from Harry; but though it appeared to him that nothing was real but the pain he was suffering, he felt a dull vexation that Miss Mountstephen should learn that he had fallen with his horse.

(*To be continued.*)

